# Impact of Stimulation Duration in taVNS—Exploring Multiple Physiological and Cognitive Outcomes

**DOI:** 10.3390/brainsci14090875

**Published:** 2024-08-29

**Authors:** Till Bömmer, Luisa M. Schmidt, Katharina Meier, Julius Kricheldorff, Heiko Stecher, Christoph S. Herrmann, Christiane M. Thiel, Kathrin Janitzky, Karsten Witt

**Affiliations:** 1 Department of Neurology, Carl von Ossietzky University, 26121 Oldenburg, Germany; 2 University Clinic for Neurology at the Evangelical Hospital, 26121 Oldenburg, Germany; 3 Experimental Psychology Lab, Department of Psychology, Carl von Ossietzky University, 26129 Oldenburg, Germany; 4 Research Center Neurosensory Science, Carl von Ossietzky University, 26129 Oldenburg, Germany; 5 Biological Psychology Lab, Department of Psychology, Carl von Ossietzky University, 26129 Oldenburg, Germany

**Keywords:** transcutaneous auricular vagus nerve stimulation (taVNS), neuromodulation, locus coeruleus (LC), stimulation duration, outcome parameters

## Abstract

Transcutaneous auricular vagus nerve stimulation (taVNS) is a non-invasive neuromodulation technique that modulates the noradrenergic activity of the locus coeruleus (LC). Yet, there is still uncertainty about the most effective stimulation and reliable outcome parameters. In a double blind, sham-controlled study including a sample of healthy young individuals (*N* = 29), we compared a shorter (3.4 s) and a longer (30 s) stimulation duration and investigated the effects of taVNS (real vs. sham) on saliva samples (alpha amylase and cortisol concentration), pupil (pupillary light reflex and pupil size at rest) and EEG data (alpha and theta activity at rest, ERPs for No-Go signals), and cognitive tasks (Go/No-Go and Stop Signal Tasks). Salivary alpha amylase concentration was significantly increased in the real as compared to sham stimulation for the 30 s stimulation condition. In the 3.4 s stimulation condition, we found prolonged reaction times and increased error rates in the Go/No-Go task and increased maximum acceleration in the pupillary light reflex. For the other outcomes, no significant differences were found. Our results show that prolonged stimulation increases salivary alpha-amylase, which was expected from the functional properties of the LC. The finding of longer response times to short taVNS stimulation was not expected and cannot be explained by an increase in LC activity. We also discuss the difficulties in assessing pupil size as an expression of taVNS-mediated LC functional changes.

## 1. Introduction

The vagus nerve (VN) is the most widespread cranial nerve in the body, and its stimulation is used for the treatment of a variety of clinical symptoms [1]. Vagus nerve stimulation (VNS) has been FDA-approved for managing drug-resistant epilepsy and depression (invasive VNS, iVNS) [2,3] and the treatment of migraine and cluster headache (transcutaneous cervical VNS, tcVNS) [4]. Transcutaneous auricular VNS (taVNS) shows potential; however, it is still under investigation for clinical application. Overall, due to their non-invasive and easy-to-use nature, tcVNS and taVNS have been widely investigated for their application in different clinical fields and for neuro-cognitive and physiological research in healthy populations [5]. In taVNS, the latter is especially crucial since there is uncertainty about effective stimulation and reliable outcome parameters [6]. In taVNS, the VN and associated regions are stimulated transcutaneously by targeting specific regions of the outer ear, most importantly the cymba conchae [7]. This area is almost exclusively innervated by the (mainly afferent) auricular branch of the vagus nerve [8] which projects to the locus coeruleus (LC) via the solitary nucleus [9]. The LC is the main source of norepinephrine (NE) in the brain. NE is crucial for attention and arousal [10]. Direct evidence for the effects of VNS on the LC-NE system stems from studies on rodents. Hulsey and colleagues (2017), for instance, found that VNS drives LC-neuron firing rates in rats [11]. Furthermore, in mice, iVNS led to an increased arousal state and an activation of subcortical structures, which, in turn, led to a widespread activation of cholinergic and noradrenergic modulatory pathways [12]. In humans, the activation of afferent fibers of the VN [13], the nucleus of the solitary tract and the locus coeruleus by taVNS has been demonstrated indirectly using fMRI [14,15].

Enhanced activity of the LC and the increase in NE cannot be measured directly in humans; therefore, indirect measures for increased NE have been used. At the brainstem level, salivary alpha amylase (sAA) concentration is an indicator for effects on the LC-NE system. The LC modulates the autonomic control of the salivation by its projections to the salivary glands: it inhibits the parasympathetic and activates the sympathetic system [16,17]. This results in an amplified secretion of sAA; thus, an increase in sAA concentration in the saliva is expected. While there are taVNS studies that have found an increase in sAA [18,19], others were not able to replicate this finding [20,21]. Saliva cortisol is considered a sensitive indicator of the stress axis and the central noradrenergic concentration [18,20,22,23] that is directly modulated by the LC. Correlations between sAA and saliva cortisol concentrations have been previously found [24,25,26] demonstrating the impact of the noradrenergic system on both markers [24]. Only two studies have investigated the cortisol level during taVNS [18,20]. In both studies, a slower cortisol concentration decrease during stimulation was found in the taVNS group compared to the sham group in post hoc analyses.

Pupil size is a non-invasive, easily measurable biomarker that indicates rapid neuromodulatory changes [27,28,29] and is directly modulated by the LC-NE system. The LC-NE system activates the sympathetic and inhibits the parasympathetic activity of the autonomous nervous system [30,31]. As the dilator muscle of the pupil is innervated by the sympathetic system [32,33,34,35], an increase in pupil dilation at rest (without any alterations in luminance conditions) during taVNS is expected. In mice, pupil dilation has been found to be a sensitive readout of VNS-mediated effects on the LC-NE system [36]. Studies on humans have found mixed results regarding the pupil size at rest. It is notable, though, that shorter stimulation durations in particular [37,38,39] compared to longer durations [18,40,41] or continuous stimulation [20,42] have led to significant pupil size changes. Furthermore, there are several parameters for the pupillary light reflex (PLR) that can be investigated. The maximum acceleration and velocity are especially sensitive to parasympathetic and sympathetic changes [43,44]. Only two studies have investigated the PLR in VNS. While no changes were visible in the light reflex measures in patients with iVNS [45], another study in healthy young individuals found a reduced amplitude and delayed onset of pupil constriction in a PLR task with taVNS as compared to sham [37].

In resting-state EEG, alpha (8–13 Hz, associated with a relaxed state, mostly found occipitally) and theta (4–8 Hz, associated with cognitive control, mostly found in midline/temporal regions) frequency bands are of interest [46,47]. The activation of the LC leads to an inhibition of thalamocortical alpha generators, which results in an attenuation of occipital alpha activity. With a decrease in alpha, increased theta and gamma activity can be observed, which leads to more focused attention [48]. The up-regulated theta activity is further regarded as a marker for cognitive control [49]. While alpha activity is down-regulated by taVNS, theta activity is thought to up-regulated [48]. Studies investigating changes in resting-state EEG due to taVNS are sparse. A study by Sharon and colleagues (2021) found an attenuation of occipital alpha in the time–frequency domain [38], but a replication of this study could not reproduce these findings [39]. Furthermore, Wienke and colleagues (2023) showed an increase in midfrontal theta [37], while another study in epilepsy patients showed the opposite effect [50]. Additionally, an increased P300 amplitude has been discussed as one potential marker for noradrenergic activity [40]. Here, VNS can lead to a more pronounced positive deflection after an unexpected stimulus (e.g., No-Go signals in Go/No-Go tasks) [51]. A systematic review revealed a general trend towards changes on early components of ERP-related inhibitory tasks [52]. In a study using an oddball task, taVNS led to an increase in P3 amplitude [53], while in another study using a similar task, a larger P3b amplitude was only found in exploratory post hoc analyses [19]. Other studies using different tasks, including a response conflict [54], Go/No-Go [55], stimulus-discrimination [18] and auditory oddball task [56], did not find an effect of tVNS on the P3 amplitude.

It has been hypothesized that taVNS promotes inhibitory control by its regulation of the LC-NE and GABA-ergic systems [40,57]. A recent meta-analysis found a taVNS effect on cognitive performance with significant effects on executive function and accuracy measures [58]. More specifically, Keute et al. (2020) found enhanced global accuracy with taVNS [59], while in another study, increased adaptation to conflict in a conflict interference task was found [54]. Furthermore, taVNS enhanced response inhibition [60] or accuracy [57] in other Go/No-Go tasks. On the other side, studies did not find an effect of taVNS on aspects of executive functions, whether it was inhibitory performance and post error slowing [61] or general performance [20,42].

The results of studies up to now are heterogeneous, and no potential biomarker of taVNS-dependent stimulation has yet shown a consistent stimulatory effect of the LC-NE system. This inconsistency may be due to differences in stimulation settings [6].

There are several stimulation parameters that can be manipulated in taVNS including the intensity (mA), frequency (Hz), pulse width (µs), duty cycle (percentage of length of rectangular pulse), and duration (ON/OFF times) of the stimulation. The manipulation of these parameters can influence the stimulation outcome. Specifically, stimulation duration has been investigated more thoroughly in recent studies as there has been a shift from the widely used 30 s ON/OFF stimulation settings. For instance, short stimulation durations (0.5 s stimulation) drove LC activity in one study [11] and had the strongest effects on pupil dilation in another (10 s stimulation) [36]. More recently, the duration of stimulation has been investigated more thoroughly in humans. Sharon et al. (2021), for instance, used short 3.4 s stimulation and found changes in pupil dilation and EEG alpha activity with taVNS [38]. A recent study was able to replicate the results for pupil dilation but not for alpha activity [39]. Recently, Skora et al. (2023) were the first to compare a short (1 s) and long (30 s) stimulation using pupil dilation as outcome [62]. They found that both short and long stimulation modulated the noradrenergic system, which was indicated by increased pupil size a few seconds after stimulation onset.

Currently, there are no studies investigating the effects of short vs. long stimulation duration in taVNS on a variety of different outcome measures. The outcome parameters of this study are assigned to functional systems to investigate the effects of LC on autonomic systems (saliva, pupil response), electro physiological correlates (alpha and theta EEG) and neurobehavioral changes (Go/No-Go task and Signal Stop task) of taVNS. In our work, we aim to expand the knowledge on different stimulation durations by testing a shorter and a longer duration using a variety of outcome parameters. Our main research questions are as follows: (1) Is there an effect of taVNS on the outcome parameters in a healthy sample? (2) Is there a difference between a shorter (3.4 s) and a longer (30 s) stimulation duration?

## 2. Materials and Methods

### 2.1. Sample

Twenty-nine individuals (fourteen females) with an average age of 25 years (*SD* = 2.5 years) participated in the study. The sample size was determined using GPower [63]. We used effect sizes from Sharon et al. (2021) [38], with Cohen’s d = 0.87 for EEG and Cohen’s d = 0.62 for pupil measurements, resulting in a required sample size of 23 for EEG and 13 for pupil. Given the lack of comparable studies assessing change in sAA and cognition (response inhibition), no sample size was calculated for these measures. Therefore, a sample size of 29 individuals was chosen to provide a buffer for potential dropouts. After being informed about the purpose of the experiment, all signed a written informed consent. Participants were excluded if they reported a history of neurologic or psychiatric diseases, had active implants (e.g., pacemakers), eye diseases or eye operations, previous brain surgeries, or were pregnant. Data was recorded at the outpatient clinic of the department of Neurology at the Evangelisches Krankenhaus Oldenburg. The study was approved by the local medical ethics committee. Participants were reimbursed with EUR 10/hour for their participation.

### 2.2. Procedure

**Figure 1:**
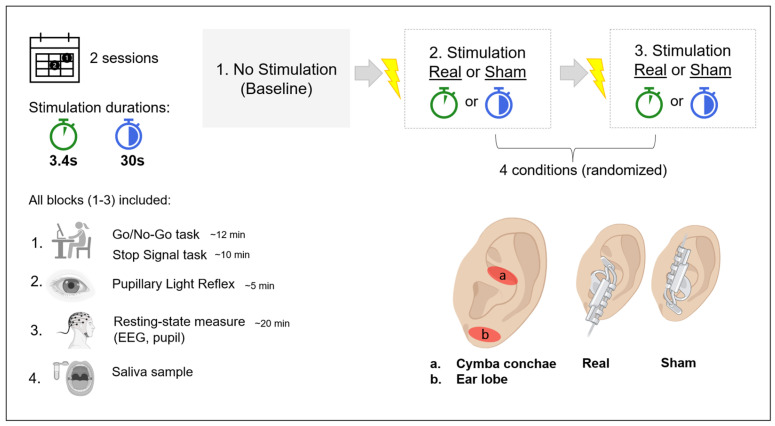
Detailed experimental procedure. Parts of the figure were created with biorender.com (accessed 21 March 2024).

The study used a randomized, sham-controlled within-subject crossover design consisting of two sessions separated by at least 48 h. A wash-out time of 48 h was selected to ensure the absence of stimulation effects from the previous session. Participants were placed on a comfortable chair in front of a screen in a dimly lit room. Before the first session, participants were asked to answer questions on demographics and initial status. At each session, participants completed three experimental blocks, each starting with a baseline block without any stimulation. The other two blocks were presented in a randomized order, either real stimulation condition or sham stimulation condition and either 3.4 s or 30 s stimulation protocol (for details please see Section 2.3). The conditions were randomized in a way that each session included one real and one sham stimulation condition. After each stimulation block, subjects were asked to indicate whether they thought that the stimulation was real or sham. Each block took approximately 45 min to complete; for details on the experimental procedure, see Figure 1.

### 2.3. Stimulation

The stimulation protocols were initiated with a Matlab script (Matlab R2020b, Mathworks, Inc., Natick, MA, USA) which sent the digital stimulation signals to a bipolar constant current stimulator (DS5 stimulator, Welwyn Garden City, UK) via a digital-to-analog converter (USB-6341 DAQ; National Instruments, Austin, TX, USA). To connect the DS5 to the ear electrode, a custom adapter (converting 3.5 mm jack to double USB) was built by the electronics workshop at the University of Oldenburg. Before each stimulation block, the individual stimulation intensity was obtained through a method of limits in which the intensity was gradually increased (starting at 0.1 mA) until the participants indicated the stimulation to be painful. The stimulation intensity that was applied before reaching the pain threshold was used for the stimulation during the experiment.

We used a legacy ear electrode (NEMOS^®^, Cerbomed, Erlangen, Germany) which consists of two titan electrodes mounted to a gel frame. The electrode was held in place with an earpiece secured with leucotape, and electrode gel was applied to the electrodes to reduce impedance. The DS5 uses a variable current (constant voltage in tissue), and we stimulated the afferent VN fibers. The real stimulation condition was applied at the cymba conchae and the sham stimulation at the ear lobe of the left ear. Stimulation parameters were as follows: frequency (25 Hz), pulse width (250 µs), pulse form (rectangular), duty cycle (0.625% on period), intensity (adjustable individual intensity, mA). In the short stimulation protocol, stimulation was on for 3.4 s and off for 26 s, as initially used by Sharon et al. (2021) [38]. The long stimulation protocol followed the standard settings of the NEMOS device, a 30 s ON/OFF duration.

### 2.4. Main Outcome Parameters

#### 2.4.1. Saliva

Saliva samples were taken during the 20 min resting-state measure (between minute 15 and 17) at the end of each measurement block. Participants were asked to place a cotton swab (Salivette, Sarstedt, Rommelsdorf, Germany) into their cheek and put it into the designated container after two minutes. The saliva samples were immediately stored in a freezer at −24 degrees Celsius. After the study was completed, all saliva samples were sent to an independent laboratory (Dresden Lab Service GmbH, Dresden, Germany). This laboratory used a measurement method by Rohleder et al. (2006) to determine the concentration of salivary alpha amylase and cortisol and the weight of the samples [64]. It is important to consider that the sAA concentration is only considered a reliable indicator if it is not affected by the change in salivary flow rate during measurement [65]. Therefore, to validate the result, it is useful to calculate the sAA secretion, which is independent of the salivary flow rate [18,24,65,66].

#### 2.4.2. Pupil

The eye tracker platform PupilCore [67] from PupilLabs was used for the detection of the pupil size at rest and the pupillary light reflex. Data was recorded with a frequency of 120 Hz and analyzed separately for the left and right eye with the 3d PupilLabs data collection method [68]. For more detail on the 3d method, see https://docs.pupil-labs.com/core/developer/pye3d/#pye3d-pupil-detection (accessed on 13 January 2023).

Pupil at rest. Participants were asked to look at a white fixation cross in the middle of the screen for 20 min. For all pupil size preprocessing steps, Python scripts in the Jupiter Notebook tool [69] were created. The guideline from Mathot et al. 2022 was considered as a basis [70] and further methodological recommendation were considered [71,72,73]. The preprocessing steps included the following: (1) cutting data in replicates (30 s stimulation: 2 s baseline, 30 s stimulation, and 28 s post-stimulation time; 3.4 s stimulation: 2 s baseline, 3.4 s stimulation, and 26.6 s post-stimulation); (2) visualization of data; (3) calculating median absolute deviation and excluding replicates with values above threshold (threshold was set based on the median of the differences plus a scaling factor = 6 multiplied by the median absolute deviation, MAD), values set to NaNs when threshold is exceeded, if more than 2% of data replicate is set to NaN, the set is excluded from further steps; (4) use of a specific algorithm to detect blinks [70,73]; (5) exclusion of data that was out of range of pupil size (1.5 mm to 9 mm); (6) removal of replicates that contained an extensive amount of removed data in steps 4 and 5 (threshold 5% or, before step 4 and 5, already 60% invalid data); (7) linear interpolation of remaining replicates; (8) application of a low-pass filter with rolling average of 100 data points; (9) subtractive baseline correction for normalization: all data was subtracted from baseline average of the replicate; (10) baseline correction including (a) transformation of baseline values to z-scores for standardization, (b) calculation of standard deviations for each z-scored baseline values from baseline average for every subject, and (c) exclusion of replicate if baseline value deviated more than 2.5 standard deviations from average baseline; (11) control of remaining replicate number: if less than 10% remained from this subject in this stimulation round, all data was removed for analysis; (12) visual inspection; (13) down-sampling data to 10 Hz.

**Figure 2:**
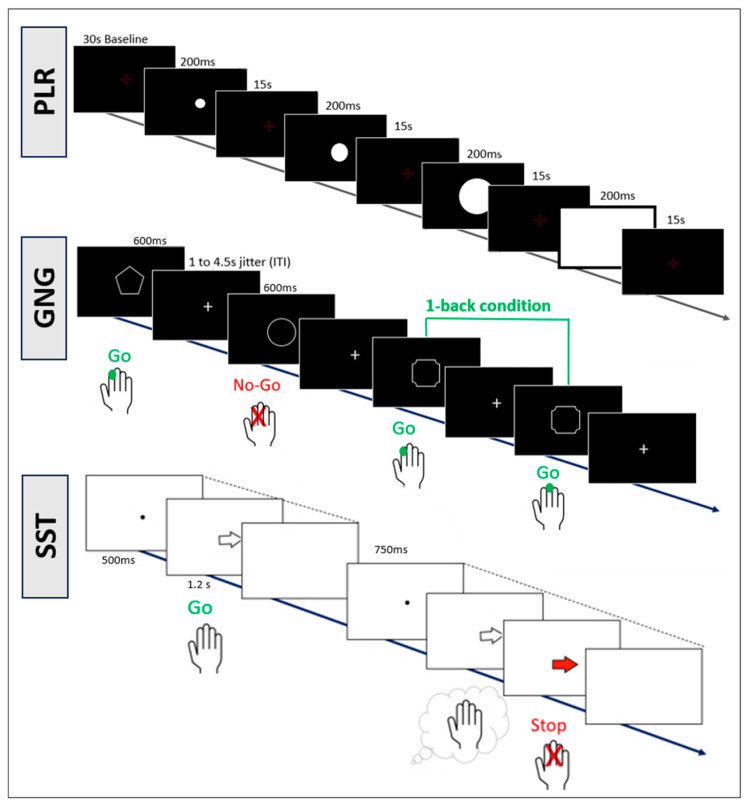
Detailed information on the three tasks in the experimental setup. Pupillary light reflex (PLR) task with different light flashes. Go/No-Go (GNG) task with 1-back condition and Stop Signal Task (SST) with stop signal after variable delay.

**Figure 3:**
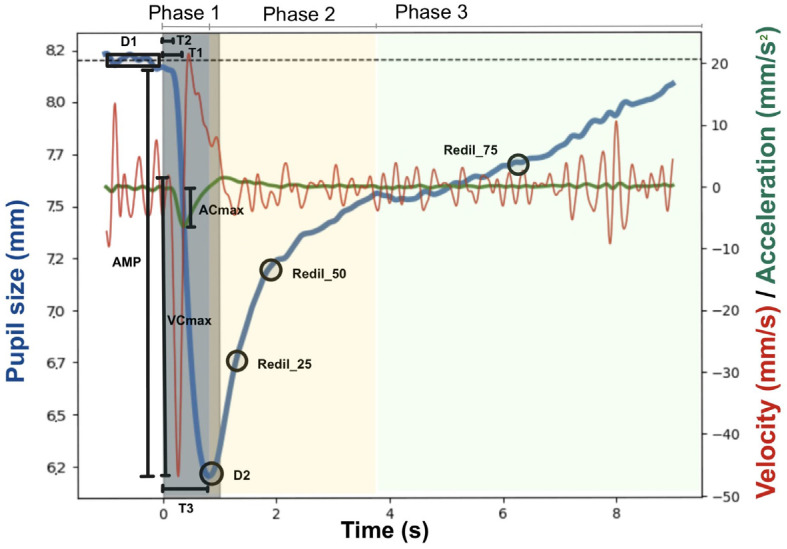
Trajectory of pupil size during pupillary light reflex and main outcomes. For more details on the parameters, please see Table A1, Appendix A.

Pupillary light reflex. The experiment included 4 light flashes with increasing size (for more details, see Figure 2 and Table A2, Appendix A) and was repeated three times (12 light flashes in total). To avoid movement artefacts and ensure constant distance to the monitor, a chin rest was used. The Python Software PyPlr (Version 1.0.2) was used as the basis and the modules utils.py and preproc.py were used for preprocessing [74,75]. The first five steps were created in the Jupiter notebook tool [69], and steps 6 and 7 with Spyder [76]. For PLR preprocessing, the following steps were included: (1) application of blink detection algorithm [70,73]; (2) visualization and control of four PLR in one data set, where if one of the criteria was not fulfilled, the data set of PLR data was excluded from further analysis: stable pupil size as baseline, rapid constriction of pupil, slow re-dilation, and no non-physiological pupillary fluctuations; (3) application of third-order low-pass Butterworth filter for remaining data sets with a cut-off frequency of 4 Hz for data smoothing; (4) division of data into separate PLRs, each starting 1 s before and 9 s after light stimulus; (5) calculation of PLR parameter with PyPlr Moduls pyplr.plr and addition of two new parameters (redil_50 and redil_25); (6) outlier removal from calculated parameters: above 3 standard deviations away from baseline value; (7) calculation of average values for each parameter for each subject. For the PLR, 12 parameters are of interest—these can be divided into measures of baseline and minimum pupil size (D1, D2), amplitude of pupil size changes (AMP, Rel_AMP), pupillary dynamics (ACmax, VCmax), time (T1, T2, T3), and time of redilation back to baseline (Redil_25, Redil_50, Redil_75. For a graphical representation of the different parameters, see Figure 3.

#### 2.4.3. EEG

EEG data were acquired using an actiChamp EEG system (actiCHamp Plus, Brain Products GmbH, Gilching, Germany) and recorded with the BrainVision Recorder software (Brain Vision Analyzer, Version 1.23.0003, Brain Products GmbH, Gilching, Germany). We recorded a 64-channel EEG with three additional electrodes to capture the stimulation artefacts. Additionally, we selected a sampling rate of 10,000 Hz to detect every single stimulation impulse. Resting-state EEG was measured simultaneously to the pupil at rest as described above. Additionally, event-related potentials (ERPs) for No-Go signals of the Go/No-Go task were recorded. For all EEG analysis steps, the MNE Python library [77,78] was used. (Pre-)processing steps of the EEG data included the following: (1) extraction of stimulation period data (only for resting-state EEG); (2) visual inspection and exclusion of EEG channels with bad quality; (3) inclusion of a reference channel; (4) re-referencing to average; (5) filter application (low-pass filer with 50 Hz and high-pass filter with 0.1 Hz); (6) down sampling (from 10,000 to 1000 Hz); (7) running Independent Component Analysis (ICA) and subsequent visual inspection of ICAs for removal of stimulation/eyeblink artefacts; (8) inclusion of an additional 25 Hz notch filter; and (9) interpolation of bad channels. After preprocessing, epochs were created for the resting-state EEG data according to the stimulation period extracted in step (1). Next, the stimulation epochs were converted into time frequency domain and averaged to obtain an averaged frequency domain over the alpha (8–13 Hz) or theta (4–8 Hz) band. This was carried out using a Morlet wavelet transform with 6 cycles and a baseline of −1 to 0 s. Lastly, the time-frequency analysis data of the real stimulation was subtracted from the data of the sham stimulation to later determine whether there were significant differences between the conditions. For ERP data, epochs were created around the No-Go stimuli from −200 to 1500 milliseconds and averaged separately for each condition (real/sham, 3.4/30 s) to extract P300 peak amplitudes of channel Pz.

#### 2.4.4. Cognitive Tasks

Two cognitive tasks were used as a measure for inhibition control. First, a modified version of the CARIT Go/No-Go (GNG) task from the Human Connectome Project was used [79]. This GNG task contained 4 Go and 2 No-Go conditions. The task consisted of three blocks of 92 trials (2/3 Go conditions). To increase difficulty, a 1-back condition was added. Participants were asked to remember the previous stimulus and decide whether it was the same as the current stimulus or not. In trials where two identical Go-stimuli occurred in succession, participants were instructed to press the left button with their index finger. In trials where a Go stimulus that was different from the previous one was presented, the participants were asked to press the right button with their middle finger. Like in ordinary GNG tasks, participants were asked not to react to No-Go stimuli, even if two identical No-Go stimuli occurred in succession. The task duration was approximately 12 min. The task was presented in the Presentation^®^ Software (Version 23.1, Neurobehavioral Systems, Inc., Berkeley, CA, USA, www.neurobs.com).

Second, we used a stop signal task (SST) provided by Verbruggen et al. (2019). The participants were asked to distinguish between a white arrow pointing left or right. For the left arrow, participants were asked to press the left arrow key on the keyboard with their index finger. For the right arrow, they were asked to press the right arrow key with their middle finger. In “Go” trials (75% of the trials), the participants were asked to react to the arrows as quickly and accurately as possible. In trials with a stop signal (25% of trials), the arrows turned red after a variable delay (i.e., stop signal delay, SSD), prompting the individual to cancel their response. The SSD (initially set to 300 ms) is modified after each trial using a staircase tracking procedure (successful inhibition: SSD increased by 50 ms; unsuccessful inhibition: SSD decreased by 50 ms) to reach a duration that produces a 50% inhibition rate. The task consisted of four blocks of 64 trials each. The respective stimulus remained on the screen until the participant reacted by pressing a button or until 1250 ms had passed. Contrary to the latency of Go reactions, the latency of reaction inhibition cannot be directly observed since successful inhibition results in the absence of a reaction. The SST was used in addition to the GNG task to determine the latency of reaction inhibition in the form of a stop signal response time (SSRT). The task took approximately 10 min. The task was programmed in jsPsych [80] and was used in an internet browser. For more information on the task, please see Verbruggen et al. (2019) [81]. Detailed information on the experimental set-up of the tasks can be found in Figure 2.

For the GNG task, the raw output files were analyzed to obtain the values of interest ((1) accuracy: correct Go response + correct No-Go rejection divided by the total of all stimuli; (2) error rate: reaction to No-Go stimuli or incorrect reaction to 1-back/normal Go condition; (3) mean reaction times for Go-responses) per subject and block. For the SST, a R Markdown script was available and used to obtain the SSRT [81].

### 2.5. Statistical Analysis

For all outcomes, the difference between baseline and each of the four stimulation conditions (3.4 s real/sham and 30 s real/sham) was calculated (difference score = condition-baseline). This allowed comparison of the stimulation effect without influence of the difference in measurement day and time. Outlier removal was performed using the median absolute deviation (MAD) method with a threshold set to 3 times MAD. For saliva and cognitive measures, as well as the P300, paired *t*-tests or Wilcoxon tests were used to test for significant differences between conditions. In cases where a significant difference was found, the Cohen’s d [82] was calculated as an indicator for effect size. Statistical analysis of the data was conducted using Python (RStudio [83] and Spyder [76]) using the statsmodels package.

#### Additional Analyses

Saliva: First, as time of day can have an influence on sAA, an additional linear mixed model analysis was performed between the time of saliva collection and amylase concentration. The start time of the study was divided into three groups: saliva collection in the morning (7:30 until 10:30 a.m.), midday (10:35 until 13:35 a.m.) and, afternoon (13:40 until 16:30 a.m.). Post hoc comparisons were conducted for the interactions between amylase concentration at different times of day and the proportion between real and sham stimulation. A random intercept at the level of participants was added to account for random effects and variations. The calculations were performed using RStudio’s lme4 library [84]. Multiple testing correction was applied using Bonferroni method. Second, to assess the relationship between stimulation intensity (mA) and amylase concentration, a correlation analysis was conducted. The Spearman rank correlation coefficient for non-normally distributed data was computed and considered significant if *p* ≤ 0.05.

Pupil: For the pupillary light reflex, a linear mixed model analysis was conducted to examine relationships between eye side, light intensity, stimulation duration, and PLR parameters. The dependent variables were the 12 PLR parameters (described in Section 2.4.2) and the independent variable of the stimulation in comparison to sham stimulation. A random intercept at the level of participants was added to account for random effects and variations. The calculations were performed using RStudio’s lme4 library [84]. To obtain the difference between real and sham stimulation, two sum-coded contrasts between the 3.4 s and 30 s stimulation and their respective sham conditions were created. Multiple testing correction was applied using Bonferroni method.

Resting-state measures: For pupil and EEG measures, data was analyzed using cluster-based permutation tests in which a statistical threshold was used to define a datapoint as significant. Multiple significant datapoints together summarize to a cluster (cluster = datapoints that have temporal and/or spatial connections). For pupil analysis, the pre-processed and down-sampled pupil size data during stimulation was used to find clusters. Each data point of the real stimulation was subtracted from the corresponding data point at the same time point from the sham condition. This was performed individually for each averaged pupil size datapoint of each participant for each condition. For EEG analysis, the pre-processed and down-sampled alpha and theta frequency activity data during stimulation over all 64 channels was used. Similar to the pupil data, here the alpha and theta frequency data (sham–real stimulation condition) for each participant and condition were used to identify clusters.

A two-tailed cluster permutation test was conducted separately for every stimulation condition (3.4 s/30 s; additionally for pupil: left/right eye) with the mne.stats.permutation_cluster_1samp_test function from MNE-Python [77] (n_permutations = 10.000, tail = 0, alpha = 0.05). Differences between real and sham stimulation over time were analyzed using this method. Given the previously reported attenuation of EEG alpha amplitude, we suspected a decrease in alpha power band in the real stimulation conditions. Higher noradrenergic cortical innervation should result in higher theta activation.

Cognitive measures: In cases where significant differences between real and sham stimulation were found, Friedmann rank sum tests were conducted to test whether the difference can be attributed to a stimulation effect or was driven by differences in the baseline conditions. In cases where significant differences between baseline conditions were found, additional pair-wise Wilcoxon rank-sum tests were conducted to test which baseline conditions differed significantly.

## 3. Results

Out of 29 individuals, 27 successfully completed all measurements at both appointments. Two did not show up to the second appointment. In 1 of the 27, the stimulation conditions of the second appointment were incorrectly designated, leading to a repetition of one condition. Therefore, only the measures for the first timepoint were used for analysis in this case (30 s condition: *n* = 27, 3.4 s condition: *n* = 26). On average, the mean stimulation intensity was 0.46 mA (*SD* = 0.2 mA) with intensities ranging from 0.1 to 1.2 mA. For the sham stimulation condition, the average stimulation intensity (*M* = 0.47 mA; *SD* = 0.25 mA, range: 0.1–1.2 mA) was about the same as for the real stimulation (*M* = 0.45 mA; *SD* = 0.18 mA, range: 0.1–1 mA). Stimulation intensity did not significantly differ between the real and sham stimulation conditions (*W* = 114.5, *p* = 0.735). No major side effects due to taVNS were reported or observed. Distinguishing between real and sham stimulation was at chance level (53.15% correct guesses throughout the four conditions).

### 3.1. Saliva

A total of 162 saliva samples (baseline, real, and sham stimulation of the 30 s and 3.4 s stimulation conditions) were collected from 27 participants. Due to technical difficulties of calculating concentrations, 137 samples for sAA and 153 stimulation samples for cortisol could be used for analysis. Mean absolute values for saliva outcomes and their respective baseline values are displayed in Table 1.

**Figure 4:**
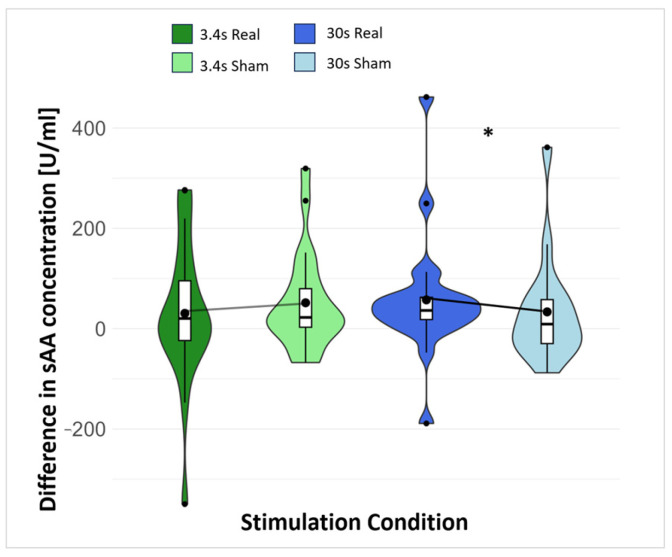
Difference score (=condition (stim/sham) − baseline) for 3.4 s and 30 s stimulation conditions for sAA concentration. sAA = salivary alpha amylase; [*] Significant difference: *p* < 0.05.

Alpha amylase: For the 3.4 s stimulation condition, no significant difference between real and sham stimulation was found for amylase concentration (*W* = 82, *p* = 0.879). For the 30 s stimulation condition, the sAA concentration was significantly increased in the real as compared to sham stimulation (*W* = 91, *p* = 0.042), with a small to medium effect size (*d* = 0.293). Additionally, for saliva secretion (absence of salivary flow rate influence), a significantly higher result was found for the 30 s real stimulation than for the 30 s sham stimulation (*W* = 98, *p* = 0.015), with a medium effect size (*d* = 0.537), indicating that the amylase concentration is slightly affected by salivary flow rate. The difference scores for 3.4 s and 30 s stimulation conditions for sAA concentration are displayed in Figure 4, and absolute scores for sAA can be found in Table 1.

The significant results of amylase concentration between 30 s real and sham stimulation were confirmed in the linear mixed model analysis (*t*(50.24) = 3.05, *p* = 0.004). Furthermore, it showed that the difference of amylase concentration during 30 s real versus sham stimulation varied depending on the time of day. A significantly lower amylase difference between real and sham stimulation was found for saliva collection in the morning in comparison to the midday amylase difference (*t*(52.14)= −2.31, *p* = 0.025). The difference in amylase concentration between real and sham during 30 s stimulation was greatest when the saliva was collected at midday (coefficients for amylase concentration difference real/sham: midday = 62.97 (*t*(50.24) = 3.05, *p* = 0.004), mornings = 2.99, afternoon = 7.72). For more detailed results, see Table A3, Appendix B. Furthermore, no significant correlations between amylase concentration and stimulation intensity were found (see Table A4, Appendix B).

Cortisol: No significant difference was found between the real and sham stimulation for either the 3.4 s (*W* = 117, *p* = 0.625) or the 30 s stimulation condition (*t*(21) = 0.163, *p* = 0.436). The difference scores for the 3.4 s and 30 s stimulation conditions for cortisol concentration are displayed in Table 1.

### 3.2. Pupil

**Figure 5:**
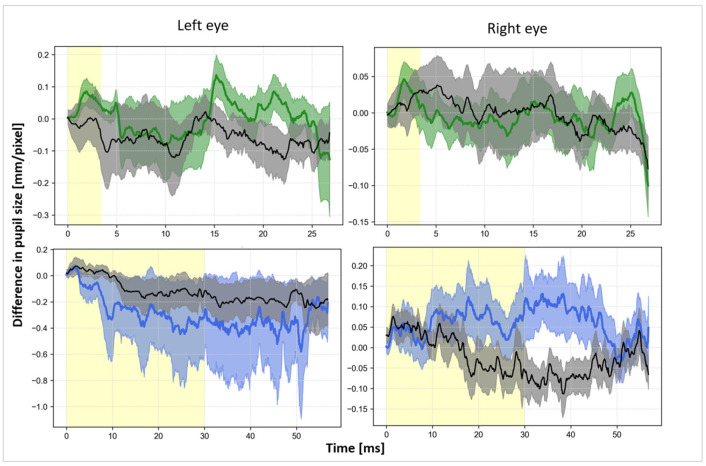
Difference scores (=condition (stim/sham) − baseline) for pupil size at rest for 3.4 s (green) and 30 s (blue) stimulation versus sham (grey) of the left and right eye. Yellow areas indicate stimulation ON time.

**Figure 6:**
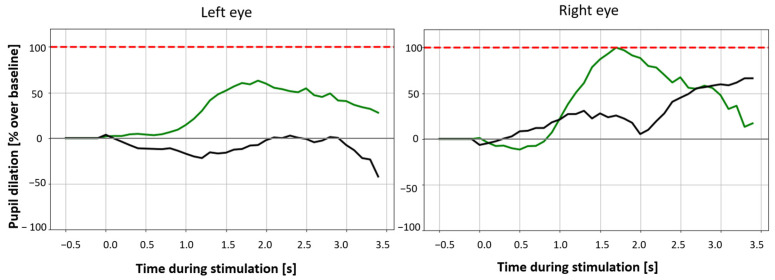
Absolute scores for pupil size at rest at stimulation onset for 3.4 s (green) and stimulation versus sham (black) of the left and right eye. The red dotted line represents the 100% threshold, while the baseline was taken as the zero point and the maximum pupil size (average of all results) as the maximum. All values are given as a percentage change from the maximum pupil size.

Pupil size rest: Real and sham stimulation revealed no significant results for pupil size at rest for either 3.4 s or 30 s conditions (permutation test, see Table A5 for detailed results). Figure 5 displays the change in difference scores of the pupil size for 30 s of the 3.4 s stimulation and for 60 s of the 30 s stimulation for left and right eye. Figure 6 demonstrates pupil size during stimulation (3.4 and 30 s). In both figures, a slight increase in pupil size at the start of the stimulation is visible for both eyes in the 3.4 s (green) condition but not in the 30 s condition.

PLR parameters: For the PLR experiment, maximum acceleration (ACmax) was higher in the real as compared to the sham stimulation for the 3.4 s condition (*t*(508.9) = 2.432, *p* = 0.038). For all other measures, no significant differences were found between real and sham stimulation for either 3.4 s or 30 s stimulation duration. Detailed results can be found in Table A6 and Table A7.

### 3.3. EEG

**Figure 7:**
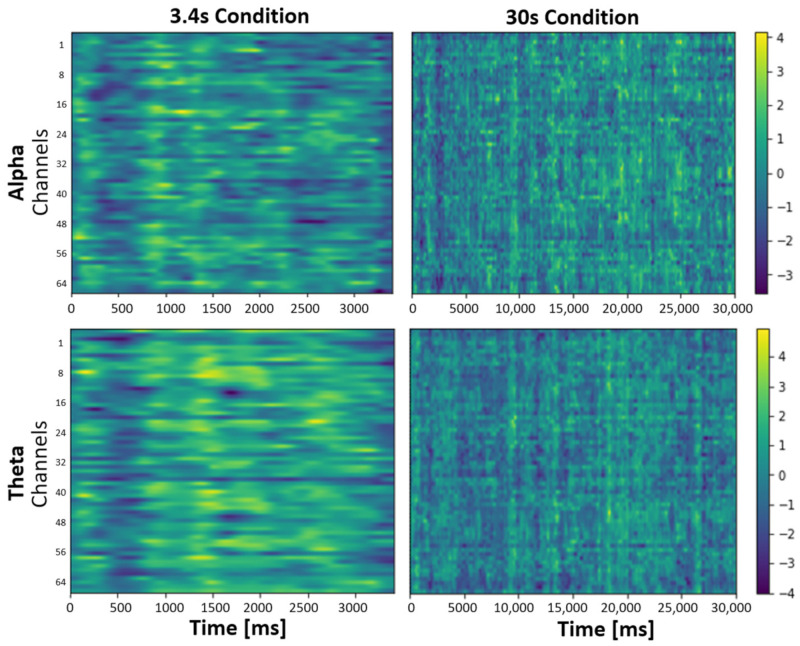
Heatmap for results of the cluster permutation test for the 3.4 s and 30 s conditions for theta and alpha frequencies during active stimulation. T-values indicated as colors (see color bar on the right side).

Alpha activity: In the 3.4 s stimulation condition, 19 clusters (alpha frequency data of 3.4 s condition, sham–real stimulation) were identified during stimulation over 64 channels. None of the identified clusters revealed significant differences between conditions (lowest value: *p* = 0.853). For the 30 s stimulation condition, 151 clusters (alpha frequency data of 30 s condition, sham–real stimulation) were identified. None of the identified clusters revealed significant differences (lowest value: *p* = 0.753). Figure 7 displays a heatmap for the alpha frequency for the 3.4 s and 30 s condition.

Theta activity: For the 3.4 s stimulation condition, 19 clusters (theta frequency data of 3.4 s condition, sham–real stimulation) were identified, and none of the clusters revealed significant differences (lowest value: *p* = 0.797). For the 30 s stimulation condition, 204 clusters (theta frequency data of 30 s condition, sham–real stimulation) were identified, and none of them revealed significant differences (lowest value: *p* = 0.677). Figure 7 displays the heatmap for the theta frequency for the 3.4 s and 30 s condition.

### 3.4. Cognition

**Figure 8:**
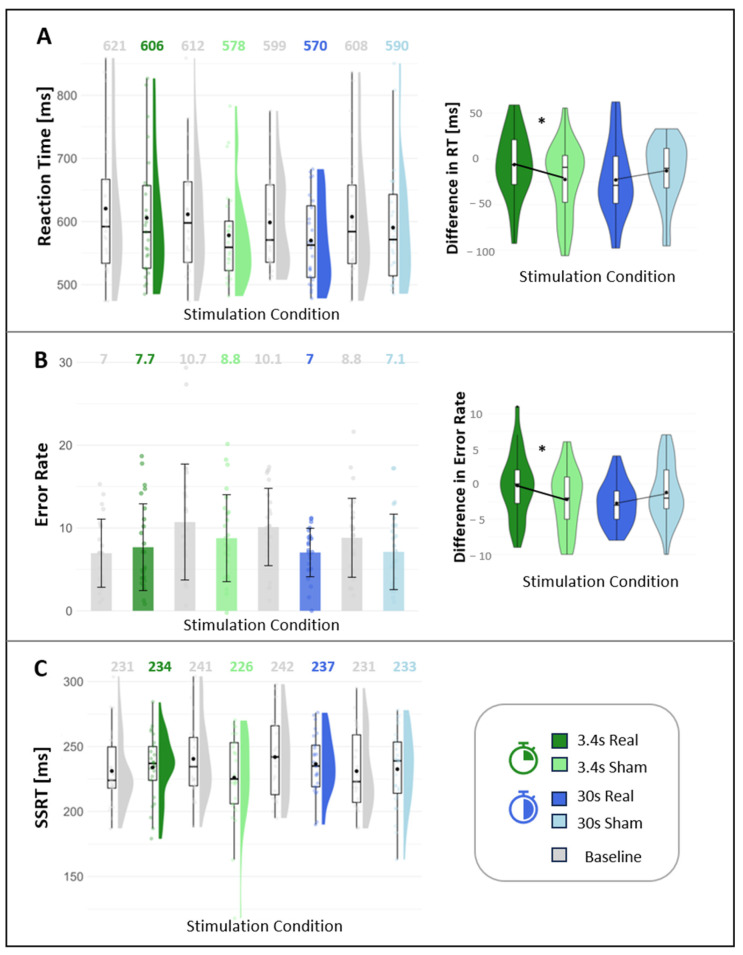
Absolute and difference scores for the Go/No-Go task (**A**,**B**) and the stop signal task (**C**) for the 3.4 s and 30 s stimulation condition and their respective baseline condition. Numbers above the plots on the left correspond to the absolute mean values. Abbr.: RT = reaction time. [*] *p* < 0.05.

Go/No-Go task: The analysis of the difference scores (condition-baseline) revealed no significant difference between real and sham stimulation for accuracy rate for either the 3.4 s (*t*(20) = −1.33, *p* = 0.196) or 30 s (*t*(22) = 1.215, *p* = 0.238) stimulation condition. The difference scores indicated a significantly increased reaction time for correct Go responses for the real stimulation as compared to sham in the 3.4 s stimulation condition (*t*(20) = 2.137, *p* = 0.045), with a medium effect size (*d* = 0.426). This was not the case for the 30 s stimulation (*t*(22) = −0.656, *p* = 0.519). Similarly, the difference scores for the error rate were significantly higher in the real as compared to sham stimulation for the 3.4 s condition (*t*(24) = 2.601, *p* = 0.016), with a medium effect size (*d* = 0.436), but not for the 30 s condition (*t*(24) = −1.502, *p* = 0.150). The comparison of baseline conditions revealed no significant differences in reaction time (χ^2^(3) = 3.5, *p* = 0.321). The baseline conditions for error rates differed significantly (χ^2^(3) = 9.5, *p* = 0.023), with significant differences between the baseline of the 3.4 s real and sham conditions (*W* = 201.5, *p* = 0.048) as well as both 3.4 s and 30 s real stimulation conditions (*W* = 183.5, *p* = 0.019). Differences in baseline performance may explain significant difference scores in the 3.4 s vs. 30 s stimulation conditions. The absolute and difference scores of the GNG task are displayed in Figure 8.

**Figure 9:**
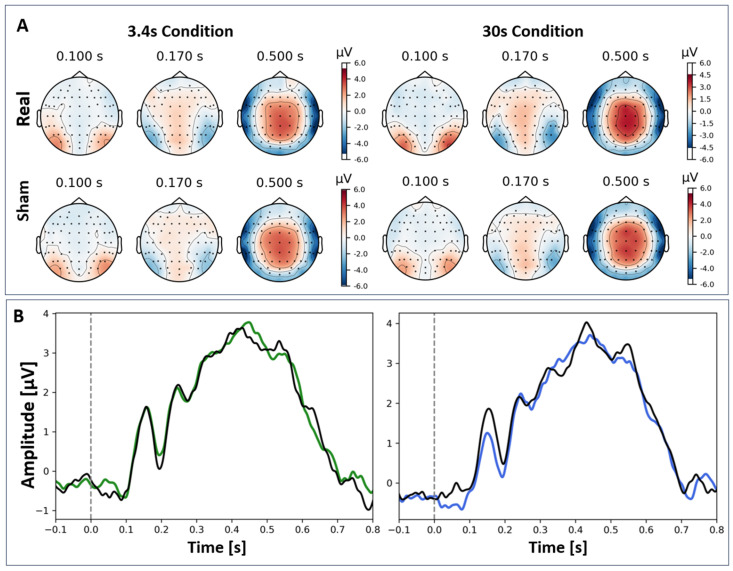
(**A**). Topoplots over all 64 channels for the four conditions. (**B**). The ERP (channel Pz) reveals multiple components including a P300 around 0.5 s (grand average) for Go stimuli in Go/No-Go task for 3.4 s (green) and 30 s (blue) conditions with their respective sham condition (black). The dotted vertical line indicates the onset of the stimulus.

P300 (No-Go signals): No significant difference between real and sham stimulation was found for P300 amplitude for either the 3.4 s (*t*(24) *=* 0.149, *p* = 0.883) or the 30 s (*t*(23) = −0.291, *p* = 0.773) stimulation condition. Figure 9 displays the average P300 for No-Go signals for the 3.4 s and 30 s conditions and their respective sham condition.

Stop Signal task: No significant differences were found between real and sham stimulation for stop signal reaction time for either the 3.4 (*t*(21) = 1.621, *p* = 0.120) or the 30 s (*W* = 143, *p* = 0.277) stimulation condition. Figure 8 displays the difference scores of the SSRT for the 3.4 s and 30 s conditions.

## 4. Discussion

The present study compared real and sham stimulation of two different stimulation durations (3.4 s vs. 30 s) and found an increased salivary alpha-amylase (sAA) concentration in the 30 s but not the 3.4 s stimulation condition. No difference in cortisol levels was found for either the 3.4 s or the 30 s condition. For pupil size at rest, no significant differences were found; however, in the pupillary light reflex (PLR), an increased maximum acceleration (ACmax) in the 3.4 s condition was found, which was not the case for the 30 s condition. Furthermore, no differences between real and sham stimulation for either the 3.4 s or 30 s condition were found for alpha and theta frequencies or for the P300 recorded during the Go/No-Go (GNG) task. Lastly, decreased performance on the GNG task (increased reaction time and error rate) was found in the real 3.4 s stimulation condition compared to the sham but not in the 30 s condition, while for the stop signal task (SST), no significant differences were found for any condition.

### 4.1. Effect of the taVNS on Saliva Marker

Our results offer interesting insights into the effects of taVNS on different physiological and behavioral outcome parameters. An increase in sAA concentration in the 30 s real stimulation condition demonstrates a neurobiological effect of the stimulation. Previous research in rodents has confirmed an effect of VNS on LC activity [11]. As sAA is seen as an indirect marker for increased LC activity, we expected and found an increase in sAA concentration as a result of a stimulation effect on the vagus nerve and the LC. Previous studies in healthy individuals have demonstrated an increased sAA concentration as well [18,19]. The stimulation effect was evident for the 30 s condition but not for the 3.4 s condition. Even though both stimulation conditions were intermittent (26 s or 30 s break without stimulation), a longer overall stimulation duration was achieved in the 30 s condition. We suspect that the longer stimulation duration is the reason for this as changes in sAA concentration occur at a slower pace than other biological processes (e.g., pupil reaction). Additionally, we found that the difference in sAA change was largest at midday. In fact, sAA levels increase throughout the day [85]. Therefore, in the context of taVNS effects on sAA concentration, it is crucial to keep the circadian rhythm of sAA in mind.

### 4.2. Effect of the taVNS on Pupil Size

Another, more dynamic indirect marker for LC activity is the pupil. In our study, a closer look at the difference in pupil size at rest is useful. There is, though it is not statistically significant, a slight difference in pupil size shortly after stimulation onset for the 3.4 s condition. This, in part, matches the results of Skora et al. (2023), who compared a shorter (1 s) and a longer (30 s) stimulation duration and found an increased pupil size in both conditions a few seconds after stimulation onset [62]. They concluded that both long and short stimulation led to phasic activation of the LC. TaVNS can modulate the activity of the LC and leads to a NE increase. The LC exhibits two different activity patterns—a phasic and a tonic mode. The phasic mode is described as short bursts of activity and is linked to facilitation of task-relevant behavior [10]. The tonic mode, on the other side, is seen as a baseline activity, and it has been proposed that it is rather linked to exploration of alternative task-related behaviors [12]. In our study, this could also be indicative of a phasic activation in the 3.4 s real stimulation condition; however, this is only speculative and needs to be taken with caution. For the 30 s condition, such an effect was not visible in our study. Other studies were not able to find an effect of taVNS on pupil size at rest [20,40,41]; however, in post hoc analyses, Capone et al. (2021) revealed that taVNS induces pupil dilation only with specific stimulation intensities (i.e., 2 mA) [86]. To increase our understanding of change in pupil size at rest in taVNS, further studies should define the optimal stimulation settings to induce a stimulation effect on the pupil.

We found an increase in maximum acceleration in the PLR in the 3.4 s stimulation condition for the real as compared to the sham stimulation. This finding is contrary to what would be expected, because the LC increases the activity of the dilator pupillae muscle and inhibits the effects of the sphincter muscle, leading to a decreased ACmax [87]. It is important to mention that in our study, the stimulation ON/OFF (3.4 s/26 s or 30 s/30 s) cycle and the light stimuli of the PLR did not necessarily occur at the same time as we did not link the stimulation “ON” time with the occurrence of PLR light stimuli. This means that possibly short-lasting effects of taVNS on the pupillary light reflex might have not been discovered since stimulation took place without the presence of a light stimulus. Linking stimulation and light stimuli in the PLR might be more insightful, which is what a recent study did [37]. They found that phasic taVNS (short stimulation duration, i.e., 500 ms) significantly increased pupil dilation and reduced the amplitude and delayed the onset of the pupil constriction of the PLR task. The authors argued that this shorter stimulation might lead to an amplification of phasic LC activity [37]. Apart from ACmax, in none of the other PLR parameters was a significant difference found in our study. The PLR is a complex process (i.e., three phases with changing (para-)sympathetic innervation), which might explain why it has barely been used in previous taVNS studies. Future studies should follow Wienke et al. (2023) and investigate the direct effects of taVNS on the pupillary light reflex by synchronizing stimulation “ON” time and light stimuli to obtain a deeper insight into dynamic changes in pupil size.

### 4.3. Effect of taVNS on EEG

A previous study by Sharon et al. (2021) reported an attenuation of alpha amplitude after taVNS stimulation onset [38]. A replication of the study was not able to confirm these results [39], which was also the case in our study for both short and long simulation conditions. Several factors apart from taVNS can influence alpha activity, for instance, time of day (EEG-derived network-alterations are more pronounced in the afternoon than in the morning [88]), the luminance in the lab (low light intensity leads to increase in alpha activity [89]), or overall vigilance of participants (leading to an increase in alpha activity [90]). Additionally, in our experiment, the resting-state measurement took place at the end of each measurement block and lasted for 20 min. It is plausible that this lengthy measurement also influenced alpha activity itself and perhaps therefore counteracted potential taVNS effects. Lastly, there are only a few studies investigating the effect of taVNS on changes in alpha activity [91,92,93,94] and none on theta activity. More studies investigating the usefulness of alpha and theta activity in the brain as markers for changes due to taVNS are needed.

### 4.4. Effect of the taVNS on Cognition

With taVNS, an enhancing effect on inhibitory control due to activation of the LC-NE system and regions in the prefrontal cortex would be expected [40,57]. However, in this study, no enhancing effect of the stimulation was visible for short or long stimulation conditions in the cognitive tasks or the P300. While there is evidence for an enhancing effect of taVNS on cognition [58], other previous studies investigating different cognitive tasks were also unable to find an stimulation effect [20,42,61]. Unexpectedly, in our study, a decrease in performance (indicated by increased reaction time and error rate) for the 3.4 s condition in the real (vs. sham) stimulation was found. Visual inspection and additional analysis of the baseline data revealed that the significant difference in the 3.4 s stimulation condition can most likely be attributed to baseline changes rather than the stimulation itself. The respective baseline conditions of the 3.4 s conditions (real, sham) differed. The error rate in the 3.4 s sham baseline condition was significantly higher than the 3.4 s real baseline condition. This most likely led to the difference scores indicating lower error rates in the 3.4 s sham condition compared with the real stimulation. For reaction times, these baseline differences were not statistically significant; therefore, an effect of taVNS is plausible. As D’Agostini et al. (2021), who also found decreased performance with taVNS, argued [20], it is plausible that suboptimal stimulation parameters can partially explain the results.

### 4.5. Differences between Invasive and Non-Invasive VNS

Previous studies have investigated the effects of iVNS across various measurements, some of which are also evaluated in the present study. The comparative outcomes of iVNS and tVNS studies are presented in the Appendix C (Table A8). Several factors may account for the observed differences in results; these include study population (e.g., patients suffering from epilepsy or depression vs. healthy controls), stimulation parameters (e.g., ON/OFF Stimulation 30 s and 5 min in patients groups vs. 3.4 and 30 s stimulation protocols in experimental studies), habituation effects (e.g., long-term stimulation in patients vs. short term stimulation in taVNS studies), and specific stimulation protocols, which may influence tonic LC functions in iVNS and phasic LC functions in taVNS. These differences have been highlighted by showing that iVNS effects depend on disease-inherent factors such as seizure frequency and stimulation duration in patients suffering from epilepsy.

### 4.6. Limitations and Future Directions

Our study brings novel insights into the effect of taVNS duration on various outcome parameters. Still, some limitations in our study and current challenges in taVNS research need to be discussed. One of the major challenges in taVNS research is the identification of optimal stimulation parameters and outcomes that reliably capture taVNS effects. Stimulation parameters can have a large influence on the stimulation outcome in taVNS. In the current study, we applied a relatively low average stimulation intensity (0.46 mA) compared to most previous studies. Helmers et al. (2012) ran a computational model on the stimulation of myelinated vagus nerve fibers and found that stimulation intensities between 0.75 and 1.75 mA could lead to optimal stimulation outcomes [95]. Possibly, the stimulation intensity used in our study was not high enough to effectively stimulate the VN and thus the LC, especially with a shorter stimulation duration. Interestingly, those studies that used stimulation intensities comparable to ours also either found no change with taVNS in any parameter or only found an increase in sAA with 30 s stimulation conditions [18,20].

Next, it needs to be noted that in our study, the quality of the pupil data was limited due to technical difficulties with the eyetracker. Additionally, the eyetracker recorded data with a sampling rate of 120 Hz; higher sampling rates (e.g., 1000 Hz) are preferred as they often ensure increased data quality. It is therefore possible that taVNS effects on the pupil remained undiscovered. Lastly, one important point to raise is the luminance during the experiment. The experiment was conducted in a dimly lit room; thus, overall, the luminosity was low. The luminance in the lab can have a large influence on outcomes such as the pupil size at rest, alpha and theta activity, cognitive tasks, and ERPs. For instance, Capone et al. (2021) only found an effect of taVNS on pupil size in a dim light condition (i.e., 0.04 Lux) [86]. Furthermore, it is known that alpha activity increases in low-light environments [96]. Since taVNS is expected to have the opposite effect, it is plausible that this factor might have counteracted potential stimulation effects in our study. In sum, luminance in the lab is an important factor and should be given special consideration in taVNS research.

## 5. Conclusions

Invasive VNS has already led to promising therapeutic effects (e.g., in epilepsy and depression), and evidence for effects of taVNS are increasing. Currently, the main challenge in taVNS research lies in the uncertainty surrounding effective stimulation parameters and reliable outcomes as indirect markers for LC-NE activity [6]. Many efforts have been made in recent years to improve standardization of taVNS studies and thoroughly investigate different stimulation parameters [40,97]. Our work provides some evidence for the effects of taVNS on tonic and phasic LC activity in a healthy young sample. There is need for more research to further explore the different effects of stimulation duration on phasic and tonic LC activity and possibly different dose–response curves for selected outcome parameters. After characterizing the optimal taVNS parameter in healthy participants, clinical trials using taVNS are necessary to investigate the impact of taVNS in disease demonstrating impaired LC function (Lewy body disease, Parkinson’s disease, Alzheimer disease). Initial studies in this area have shown a possible indication for the use of VNS to treat freezing of gait in PD [98].

## Figures and Tables

**Table 1:** Mean absolute scores for saliva outcomes with respective baseline scores. Note: SD in brackets; sAA = salivary alpha amylase; sAA concentration in U/mL, sAA secretion in U/min, cortisol concentration in nmol/L.

	3.4 s Real Stimulation (Baseline)	3.4 s Real Stimulation	3.4 s Sham Stimulation (Baseline)	3.4 s Sham Stimulation	30 s Real Stimulation (Baseline)	30 s Real Stimulation	30 s Sham Stimulation (Baseline)	30 s Sham Stimulation
sAA concentration (U/mL)	130.43 (160.05)	160.96 (142.26)	90.95 (76.10)	142.52 (91.31)	95.46 (73.90)	151.93 (125.30)	98.43 (56.24)	123.92 (112.17)
sAA secretion (U/min)	20.00 (31.74)	39.12 (55.30)	21.37 (39.87)	43.21 (50.95)	25.38 (40.47)	47.06 (67.35)	24.96 (33.64)	28.92 (45.24)
Cortisol concentration (nmol/L)	4.03 (1.72)	2.20 (0.78)	4.17 (2.06)	2.48 (1.14)	4.10 (2.06)	2.37 (1.08)	4.40 (2.23)	2.17 (1.13)

## Data Availability

The data presented in this study are available on request from the corresponding author due to technical limitations.

## Appendix A

**Table A1:** Details of pupillary light reflex (PLR) parameters. Each parameter can be assigned either to the first (parasympathetic regulated), the second (sympathetic-regulated), or the third phase (parasympathetic as well as sympathetic-regulated); P = parasympathetic, S = sympathetic.

Parameter	Unit	Description	Phase of PLR	Controlled by	Effect of LC-NE Activation on Pupil Size
D1	mm	Output variable (mean value 1 s before annotation)	1	P	larger
D2	mm	Minimal pupil size	1	P	larger
AMP	mm	Constriction amplitude: Difference between initial width and max. constriction (D1-D2)	1	P	smaller
VCmax	mm/s	Maximum constriction speed	1	P	smaller
ACmax	mm/s^2^	Maximum constriction acceleration	1	P	smaller
T1	s	Latency from light annotation to maximum acceleration (ACmax)	1	P	larger
T2	s	Latency from light annotation to maximum speed (VCmax)	1	P	larger
T3	s	Latency from light annotation to maximum constriction (D2)	1	P	larger
Rel_AMP	%	Constriction amplitude in relation to the output width (AMP/D1)	1	P/S	smaller
Redil_25	s	Time after max. constriction until pupil size reaches 25% of initial value	2	P/S	smaller
Redil_50	s	Time after max. constriction until pupil size reaches 50% of the initial value	2	P/S	smaller
Redil_75	s	Time after max. constriction until pupil size reaches 75% of the initial value	3	S	smaller

**Table A2:** Details on light intensities of PLR.

Light Stimulus Sequence	Width in Height Units ^1^	Height in Height Units	Brightness in Lux
1	0.1	0.1	0.0 ^2^
2	0.15	0.15	1.8
3	0.25	0.25	5.0
4	5.00	5.00	130.0

^1^: Height Units is a normalized unit for Psychopy, which is defined in relation to the screen size (2560 × 1440 pixels). ^2^: Brightness not detectable with digital luxmeter.

## Appendix B

**Table A3:** Post hoc analysis of the time-of-day dependence of amylase concentrations.

Condition	Contrast	Estimated Value (ß)	Standard Error	Degrees of Freedom	t-Value	*p*-Value	Adjusted *p*-Value
3.4 s	morning vs. afternoon	−13.96	22.33	52.47	−0.62	0.535	0.802
	morning vs. noon	6.66	25.07	54.68	0.27	0.792	0.847
	noon vs. afternoon	−20.61	26.50	52.33	−0.78	0.440	0.802
30 s	morning vs. afternoon	−4.73	24.44	53.51	−0.29	0.847	0.847
	morning vs. noon	−59.98	26.13	53.62	−2.30	0.026	0.154
	noon vs. afternoon	55.25	28.20	52.29	1.96	0.055	0.166

**Table A4:** Descriptive statistics of the results of the correlation analyses (Spearman) for sAA concentration separately for each condition.

	3.4 s Real	3.4 s Sham	30 s Real	30 s Sham
Sample (n)	21	22	21	16
Mean (U/mL)	19.12	21.84	24.61	7.37
Standard deviation	41.38	40.51	44.30	26.52
Shapiro-wilk (*p*-value)	0.00005	0.00018	0.00006	0.006
Correlation coefficient (r)	−0.03	−0.03	−0.42	0.17
*p*-value	0.897	0.909	0.057	0.517

## Appendix C

**Table A5:** Cluster results for pupil size.

Stimulation Duration	Eye	Number of Subjects	Threshold	Number of Clusters	*p*-Values
30 s	left	19	2.100922	0	-
	right	25	2.063899	4	0.101; 0.357; 0.214; 0.396
3.4 s	left	25	2.063899	0	-
	right	21	2.085963	0	-

**Table A6:** Mean absolute difference scores for PLR outcomes for 3d method (average of light strengths and eyes), with SD in brackets.

Parameter	3.4 s Real	3.4 s Sham	30 s Real	30 s Sham
D1	−0.323 (0.339)	−0.425 (0.164)	−0.312 (1.934)	0.541 (2.358)
D2	−0.216 (0.190)	−0.332 (0.117)	−0.268 (0.098)	−0.229 (0.147)
AMP	−0.107 (0.167)	−0.094 (0.120)	−0.092 (0.095)	−0.007 (0.086)
VCmax	0.383 (1.649)	−0.677 (0.842)	−0.722 (0.660)	0.326 (0.656)
ACmax	15.054 (31.004)	−8.031 (11.917)	−12.584 (14.293)	4.463 (11.757)
T1	−0.003 (0.060)	−0.042 (0.054)	−0.027 (0.046)	−0.008 (0.048)
T2	0.073 (0.181)	0.323 (0.386)	−0.155 (0.165)	0.000 0.283
T3	0.074 (0.174)	−0.021 (0.065)	−0.224 (0.264)	−0.006 (0.188)
rel_AMP	0.001 (0.018)	0.010 (0.022)	0.001 (0.018)	0.008 (0.014)
redil_75	−0.214 (0.544)	−0.080 (0.343)	0.015 (0.458)	0.038 (0.251)
redil_50	−0.079 (0.164)	−0.001 (0.060)	−0.033 (0.094)	0.010 (0.089)
redil_25	−0.039 (0.037)	−0.010 (0.030)	−0.007 (0.037)	0.012 (0.057)

**Table A7:** Results of linear mixed model analysis for pupillary light reflex.

Independent Variables	Parameter	Estimated Value (ß)	Standard Error	Degrees of Freedom	t-Value	*p*-Value	Adjusted *p*-Value
ACmax	(Intercept)	−7.65	6.86	35.65	−1.11	0.272	0.272
	3.4 s real vs. sham	20.358	8.372	508.897	2.432	0.015	0.038
	30 s real vs. sham	−15.72	8.23	517.33	−1.91	0.057	0.094
	Light strength (Lux)	−0.08	0.05	502.07	−1.46	0.145	0.181
AMP	(Intercept)	−0.10	0.06	27.34	−1.80	0.082	0.335
	3.4 s real vs. sham	−0.03	0.06	507.42	−0.45	0.656	0.656
	30 s real vs. sham	−0.05	0.06	517.80	−0.82	0.414	0.518
	Light strength (Lux)	−0.0005	0.0004	500.56	−1.25	0.210	0.351
rel_AMP	(Intercept)	0.002	0.01	42.50	0.21	0.832	0.832
	3.4 s real vs. sham	−0.01	0.01	514.15	−1.07	0.286	0.715
	30 s real vs. sham	−0.01	0.01	521.71	−0.73	0.466	0.716
	Light strength (Lux)	−0.00004	0.00007	506.07	−0.56	0.573	0.716
D1	(Intercept)	−0.89	0.44	70.17	−2.01	0.048	0.102
	3.4 s real vs. sham	0.14	0.72	519.32	0.19	0.848	0.851
	30 s real vs. sham	−0.13	0.71	523.91	−0.19	0.851	0.851
	Light strength (Lux)	0.01	0.005	510.57	2.37	0.018	0.090
D2	(Intercept)	−0.44	0.14	19.70	−3.04	0.007	0.033
	3.4 s real vs. sham	0.13	0.09	501.39	1.434	0.152	0.379
	30 s real vs. sham	0.08	0.09	508.47	0.93	0.355	0.592
	Light strength (Lux)	0.00003	0.0006	499.07	0.05	0.964	0.964
VCmax	(Intercept)	−0.58	0.38	37.08	−1.52	0.137	0.172
	3.4 s real vs. sham	0.95	0.47	511.55	2.04	0.042	0.069
	30 s real vs. sham	−0.94	0.46	519.68	−2.05	0.041	0.069
	Light strength (Lux)	−0.002	0.003	504.94	−0.72	0.469	0.469
Redil_25	(Intercept)	0.001	0.02	35.22	0.06	0.954	0.954
	3.4 s real vs. sham	−0.03	0.02	509.68	−1.45	0.147	0.367
	30 s real vs. sham	−0.01	0.02	518.35	−0.62	0.533	0.781
	Light strength (Lux)	0.00006	0.0001	502.70	0.49	0.625	0.781
Redil_50	(Intercept)	0.06	0.05	32.13	1.004	0.323	0.404
	3.4 s real vs. sham	−0.09	0.06	510.40	−1.39	0.164	0.379
	30 s real vs. sham	−0.01	0.06	519.23	−0.25	0.805	0.805
	Light strength (Lux)	−0.00045	0.0004	504.52	−1.21	0.228	0.379
Redil_75	(Intercept)	0.07	0.15	41.16	0.44	0.661	0.964
	3.4 s real vs. sham	−0.15	0.21	513.67	−0.70	0.481	0.964
	30 s real vs. sham	0.01	0.21	521.54	0.045	0.964	0.964
	Light strength (Lux)	−0.003	0.001	504.89	−2.43	0.02	0.08
T1	(Intercept)	0.01	0.02	37.61	0.59	0.556	0.556
	3.4 s real vs. sham	0.04	0.02	511.60	1.62	0.106	0.177
	30 s real vs. sham	−0.01	0.02	519.70	−0.66	0.511	0.556
	Light strength (Lux)	−0.0003	0.0001	504.22	−1.84	0.0677	0.167
T2	(Intercept)	0.008	0.08	515.00	0.10	0.924	0.924
	3.4 s real vs. sham	−0.28	0.16	515.00	−1.79	0.073	0.249
	30 s real vs. sham	−0.16	0.15	515.00	−1.08	0.279	0.348
	Light strength (Lux)	−0.001	0.001	515.00	−1.12	0.263	0.348
T3	(Intercept)	−0.06	0.09	37.53	−0.61	0.547	0.571
	3.4 s real vs. sham	0.07	0.11	502.80	0.65	0.515	0.571
	30 s real vs. sham	−0.22	0.11	510.76	−2.01	0.045	0.224
	Light strength (Lux)	0.0008	0.0007	496.83	1.11	0.270	0.571

**Table A8:** Comparison of studies using invasive (iVNS) and non-invasive (nVNS) vagus nerve stimulation.

Study	iVNS	nVNS
Alpha Amylase		
Doerr et al., 2023 [99] 10.1002/epi4.12774 (Patients with epilepsy)	-	No effect of tVNS on sAA activity
Warren et al., 2019 * 10.1016/j.brs.2018.12.224 (Healthy individuals)	-	taVNS increased sAA
Venutra-Bort et al., 2018 * 10.3389/fnhum.2018.00202 (Healthy individuals)	-	taVNS increased sAA levels after stimulation
D’Agostini et al., 2021 * 10.1111/psyp.13885 (Healthy individuals)	-	taVNS did not increase sAA
D’Agostini 2023 * 10.1016/j.biopsycho.2023.108646 (Healthy individuals)	-	TaVNS did not increase sAA
D’Agostini et al., 2022 * 10.1111/psyp.13984 (Healthy individuals)	-	No effect on sAA secretion with taVNS
Giraudier et al., 2020 [100] 10.3389/fpsyg.2020.01276 (Healthy individuals)	-	No tVNS effects on sAA level changes
Koenig et al., 2019 [101] 10.1017/S0033291719003490 (Adolescents with depression)	-	No effect of tVNS on sAA
Pupil Response		
Vespa et al., 2022 [102] 10.1016/j.brs.2022.11.002 (Patients with epilepsy)	iVNS induced late PDR	-
Desbaumes Jodoin et al., 2015 * 10.1016/j.ijpsycho.2015.10.001 (Patients with epilepsy and depression)	iVNS induced PDR	-
Skora et al., 2024 * 10.1101/2023.09.20.558607 (healthy controls)	-	taVNS induced early PDR
D’Agostini et al., 2023 [103] 10.1016/j.cortex.2022.11.012 (Healthy individuals)	-	Increase in event-related pupil dilation with taVNS
Lloyd et al., 2023 * 10.1016/j.brs.2023.06.010 (Healthy individuals)	-	taVNS induced early PDR
Sharon et al., 2021 * 10.1523/JNEUROSCI.1361-20.2020 (Healthy individuals)	-	taVNS induced early PDR
Urbin et al., 2021 [104] 10.1016/j.brs.2021.06.002 (Healthy individuals)	-	taVNS induced early PDR
Villani et al., 2023 [105] 10.1016/j.psyneuen.2022.105719 (Healthy individuals)	-	Event-related taVNS decreased pupil dilation in response to target stimuli
Borges et al., 2021 * 10.1016/j.ijpsycho.2021.01.003 (Healthy individuals)	-	No effect on pupillary responses
Burger et al., 2020 * j.biopsycho.2020.107863 (Healthy individuals)	-	tVNS did not affect pupil dilation
Capone et al., 2021 * 10.1016/j.clinph.2021.05.014 (Healthy individuals)	-	taVNS induced pupil dilation under specific luminance and stimulation intensity
D’Agostini et al., 2021 * 10.1111/psyp.13885 (Healthy individuals)	-	taVNS did not increase pupil size
D’Agostini et al., 2022 * 10.1111/psyp.13984 (Healthy individuals)	-	taVNS did not impact event-related pupil dilation
Keute et al., 2019 * 10.1038/s41598-019-47961-4 (Healthy individuals)	-	No modulation of pupil size or event-related pupil response with taVNS
Warren et al., 2019 * 10.1016/j.brs.2018.12.224 (Healthy individuals)	-	Pupil size not affected by tVNS
Wienke et al., 2023 * 10.1523/JNEUROSCI.0452-23.2023 (Healthy individuals)		Increased pupil dilation with taVNS
EEG		
Sharon et al., 2021 * 10.1523/JNEUROSCI.1361-20.2020 (Healthy individuals)	-	Attenuation of occipital alpha with taVNS
Lloyd et al., 2023 * 10.1016/j.brs.2023.06.010 (Healthy individuals)	-	No attenuation of occipital alpha
Wienke et al., 2023 * 10.1523/JNEUROSCI.0452-23.2023 (Healthy individuals)	-	Increase in midfrontal theta with taVNS
Pan et al., 2024 * 10.1111/cns.14395 (Patients with epilepsy)	-	Decrease in midfrontal theta with taVNS
Li et al., 2023 [106] 10.1089/brain.2022.0011 (Patients with epilepsy)	Patients showed reductions in functional connectivity in theta and alpha bands	-
Neuhaus et al., 2007 [107] 10.1016/j.jad.2006.10.005 (Patients with depression)	P300 was significantly increased in responders	-
De Taeye et al., 2014 [108] 10.1007/s13311-014-0272-3 (Patients with epilepsy)	Increase of P300 amplitude in VNS responders	-
D’Agostini 2023 * 10.1016/j.biopsycho.2023.108646 (Healthy individuals)	-	taVNS did not increase P3b
Ventura-Bort 2018 * 10.3389/fnhum.2018.00202 (Healthy individuals)	-	Larger P3b amplitudes during tVNS
Warren 2019 * 10.1016/j.brs.2018.12.224 (Healthy individuals)	-	P3 amplitude not affected by tVNS
Fischer 2018 * 10.3758/s13415-018-0596-2 (Healthy individuals)	-	No effect of tVNS on P3
Pihlaja 2020 * 10.3389/fnhum.2020.561780 (Healthy individuals)	-	No effect on P3 with tVNS
Gadeyne 2022 * 10.1016/j.clinph.2021.11.079 (Healthy individuals)	-	No differences in P3b amplitude or latency with tVNS
Response Inhibition		
Schevernels et al., 2016 [109] 10.1016/j.yebeh.2016.09.014 (Patients with epilepsy)	iVNS induced inhibitory benefit	-
Zhu 2023 * 10.1101/2023.09.14.23295532 (Healthy individuals)	-	Improved No-Go response accuracy with taVNS
Beste et al., 2016 * 10.1016/j.brs.2016.07.004 (Healthy individuals)	-	Better response inhibition performance with taVNS
Koenig et al., 2019 [101] 10.1017/S0033291719003490 (Adolescents with depression)	-	Altered response inhibition in emotional Go/NoGo task
Beste et al., 2016 * [60] 10.1016/j.brs.2016.07.004 (Healthy individuals)	-	Improved response inhibition performance with taVNS
Van Bochove et al., 2016 [110] 10.1016/j.ijpsycho.2018.03.015 (Patients with epilepsy)	Improved ability to suppress distractor interference with VNS	

Note. sAA = salivary alpha amylase; PDR = pupil dilation response; P3b = ERP P300, late component; * = studies included in Section 1.
